# Whole genome sequencing of an ExPEC that caused fatal pneumonia at a pig farm in Changchun, China

**DOI:** 10.1186/s12917-017-1093-5

**Published:** 2017-06-09

**Authors:** Ling-Cong Kong, Xia Guo, Zi Wang, Yun-Hang Gao, Bo-Yan Jia, Shu-Ming Liu, Hong-Xia Ma

**Affiliations:** College of Animal Science and Technology, Jilin Agricultural University, Changchun, China

**Keywords:** ExPEC, Pneumonia, Pigs, Genome

## Abstract

**Background:**

In recent years, highly frequent swine respiratory diseases have been caused by extraintestinal pathogenic *Escherichia coli* (ExPEC) in China. Due to this increase in ExPECs, this bacterial pathogen has become a threat to the development of the Chinese swine industry. To investigate ExPEC pathogenesis, we isolated a strain (named SLPE) from lesioned porcine lungs from Changchun in China, reported the draft genome and performed comparative genomic analyses.

**Results:**

Based on the gross post-mortem examination, bacterial isolation, animal regression test and 16S rRNA gene sequence analysis, the pathogenic bacteria was identified as an ExPEC. The SLPE draft genome was 4.9 Mb with a G + C content of 51.7%. The phylogenomic comparison indicated that the SLPE strain belongs to the B1 monophyletic phylogroups and that its closest relative is *Avian Pathogenic Escherichia coli* (APEC*)* O78. However, the distribution diagram of the pan-genome virulence genes demonstrated significant differences between SLPE and APEC078. We also identified a capsular polysaccharide synthesis gene cluster (CPS) in the SLPE strain genomes using blastp.

**Conclusions:**

We isolated the ExPEC (SLPE) from swine lungs in China, performed the whole genome sequencing and compared the sequence with other *Escherichia coli* (*E. coli*). The comparative genomic analysis revealed several genes including several virulence factors that are ExPEC strain-specific, such as fimbrial adhesins (*pap*G II), *ire*A, *pgt*P, *hly*F, the *pix* gene cluster and *fec*R for their further study. We found a CPS in the SLPE strain genomes for the first time, and this CPS is closely related to the CPS from *Klebsiella pneumoniae*.

**Electronic supplementary material:**

The online version of this article (doi:10.1186/s12917-017-1093-5) contains supplementary material, which is available to authorized users.

## Background

According to genetic and clinical criteria, *E. coli* has been classified into commensal, intestinal pathogenic and ExPEC groups [1]. ExPEC has become a bacterial pathogen that threatens the health of humans and animals. These strains can cause a wide range of extraintestinal infections, including in the urinary tract, central nervous system, circulatory system and respiratory system [2]. In recent years, some reports have demonstrated that ExPEC has been frequently isolated from clinical samples in the swine industry in China [3], leading to significant economic losses.

To date, several *E. coli* isolates have had their whole genomes sequenced, including intestinal pathogenic *E. coli* and extraintestinal pathogenic *E. coli*. Based on the whole sequenced genomes, *E. coli* can be classified into four major phylogroups (A, B1, D1, D2 and B2). ExPEC are mainly distributed into phylogroups B2, D1, D2 and F, but occasionally into other phylogroups such as A and B1 depending on the virulence gene repertoire [4]. However, to date, little work has been conducted to characterize the ExPEC that caused fatal pneumonia at a pig farm in Changchun, China. In this paper, we isolated the ExPEC from swine lungs from China and describe the genome characteristics of the isolate.

## Results

### Gross post mortem examination and histological examination

**Fig. 1:**
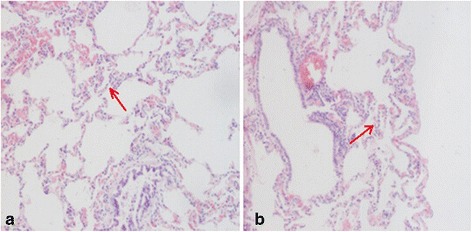
Histological preparation of swine lungs (haematoxylin and eosin). Note the presence of inflammatory cells in the bronchioles (**a**) and the surrounding alveoli (**b**)

The pig carcass was in fair nutritional condition. There was no evidence of any viruses or external parasites. Only the lungs had some congestion and bleeding. The stomach was devoid of contents. There was nothing remarkable about any of the other visceral systems. The histological preparations of the lungs showed the presence of inflammatory cells in the bronchioles and the surrounding alveoli (red arrow), and presence of some bleeding and edema (Fig. 1). Histological examinations of pneumonia-like presentation may be classified as bronchopneumonia.

### Bacterial isolates and serotyping

Some colonies were obtained from the pig lung, in colonies, only one colony was selected, which contained two of the ExPEC virulence markers, iutA and papC, named SLPE. The SLPE isolate was positive for serogroup O149.

### Experimental challenge studies

The SLPE isolate killed all of the mice at the 5 × 10^7^ CFU, 5 × 10^6^ CFU and 5 × 10^5^ CFU challenge doses, but the surviving time of each group is different, the 5 × 10^7^ CFU dose group only survived 6 to 8 h and the 5 × 10^6^ CFU and 5 × 10^5^ CFU dose group survived 12 to 18 h. All of the mice in the negative control group survived without any clinical signs. All tested were dissected and examined for lesion, the lungs had some bleeding and swelling, but there was nothing remarkable lesions in other tissues and organs. The SLPE presented as a highly virulent ExPEC strain in the mouse model. Chen et al., has isolated 315 ExPEC in china, only 2 isolated of these killed 5/5 mice at a challenge dose of 10^5^ CFU [5].

### Antimicrobial resistance

The MIC values for the 8 antimicrobial agents obtained from the examinations of the SLPE isolates are shown. Cefotaxime was found to be the most active compound in vitro (MIC < 0.03 μg/mL). The isolates was already resistant to the rest of the antimicrobials and displayed the following MIC values: (1) florfenicol, MIC = 128 μg/mL; (2) doxycycline, MIC = 128 μg/mL; (3) ciprofloxacin, MIC = 64 μg/mL; (4) tilmicosin, MIC = 512 μg/mL; (5) enrofloxacin, MIC = 128 μg/mL; (6) sulfamethoxazole, MIC>512 μg/mL and (7) amikacin, MIC = 128 μg/mL.

### Genomic features

A total of 336 contigs from the genome were assembled onto 152 scaffolds. The predicted genome size was 4.9 Mb, with an N50 value of 106,661 for the assembly. Ombining the glimmer 3.02, Genemark and Z-Curve programs annotations gave 4806 genes with approximately 51.7% GC content, which is similar to other reported *E. coli* genomes. This Whole Genome Shotgun project has been deposited at DDBJ/EMBL/GenBank under the accession LJCG00000000.1. The SLPE genome harbour some resistance genes, including MarC, SoxR, MATE, ABC genes, tet, FloR and also carrie class Iintegrons. but the gene cassettes of Iintegrons genes, that conferred resistance to tetracycline, fluoroquinolone, aminoglycosides and so on.

### Phylogenomic genome analysis of SLPE with other *E. coli* pathotypes

**Fig. 2:**
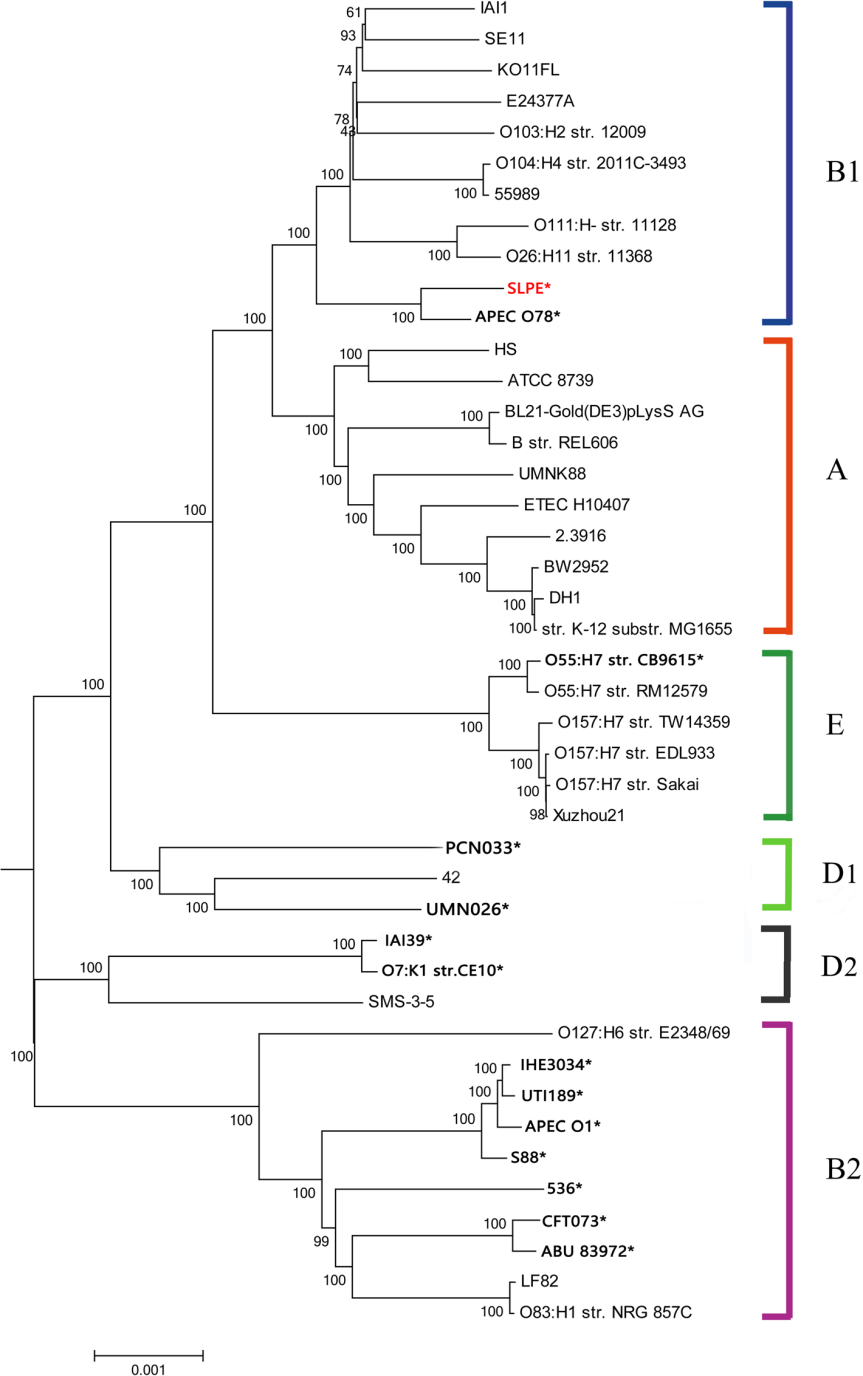
Phylogenomic tree of the 43 *E. coli* strains. Blastp, perl, mcl, clustalW and MEGA were used to reconstruct the phylogenomic tree. The phylogenomic tree of different *E. coli* based on the comparison of 2043 core genes. The 43 *E. coli* strains are divided into five monophyletic phylogroups (A, B1, B2, D and E). The SLPE strains are highlighted in blackbody. SLPE is the closest relative to APEC 078

The phylogenomic tree was constructed to examine the SLPE strain in the context of the *E. coli* population structure. Figure 2 shows that the 43 *E. coli* strains were divided into five monophyletic phylogroups (A, B1, B2, D and E). The SLPE strain belongs to the B1 monophyletic phylogroup, and its closest relative is APEC 078.

### Distribution diagram of the pan-genome virulence genes

**Fig. 3:**
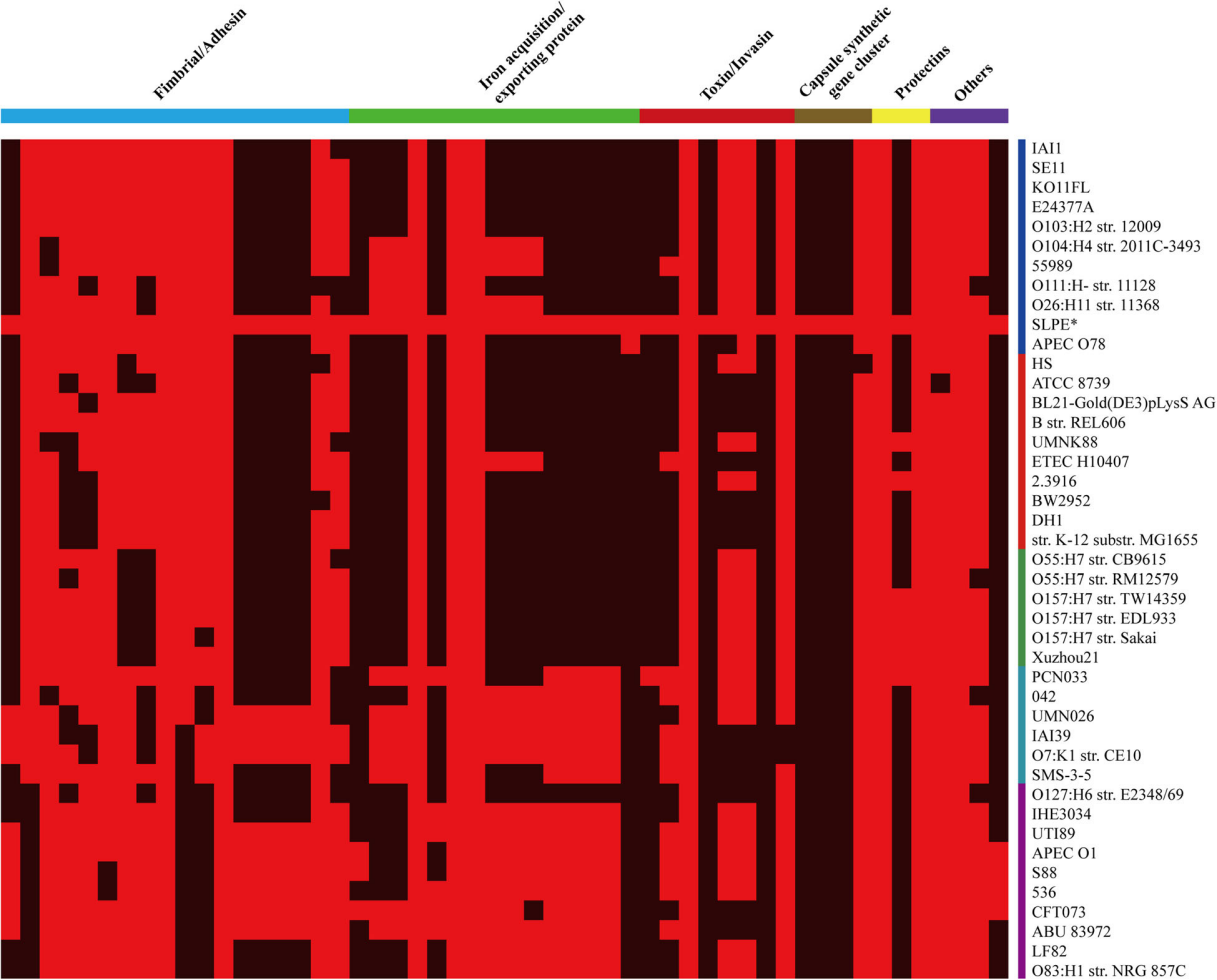
The distribution diagram of the pan-genome virulence genes among 43 *E. coli* strains. The right side of the vertical line shows *E. coli* strains that are consistent with the phylogenetic tree (Figure 2) with the following labels: (1) B1 monophyletic phylogroup, blue; (2) A monophyletic phylogroup, red; (3) E monophyletic phylogroup, green; (4) D monophyletic phylogroup, cyan and (5) B2 monophyletic phylogroup, purple. The SLPE strains are highlighted with the key symbol. The top shows the following six classified clusters: (1) fimbrial/adhesin, blue; (2) iron acquisition/exporting protein, green; (3) toxin/invasin, red; (4) capsule synthetic gene cluster, brown; (5) protectins, yellow and (6) others, purple. The red and black bodies show the distribution of the virulence genes among these strains. A red side implies that the virulence genes are present, while the black side implies that the genes are absent

The distribution of the ExPEC virulence factors were analysed among the 43 sequenced *E. coli* strains. Figure 2 shows that ExPEC-specific virulence factors were classified into the following six categories: (1) adhesins, (2) invasins, (3) toxins, (4) iron acquisition/transport systems, (5) polysialic acid synthesis and (6) other virulence genes. The 51 virulence genes from the 43 sequenced strains are shown in Fig. 3. The distribution diagram shows that even though there were significant differences between the SLPE and APEC078 strains, that these two strains are most closely related to one another. We also found that SLPE has more ExPEC-specific virulence factors, including several virulence factors that are ExPEC strain-specific, such as fimbrial adhesins (*papG* II), *ire*A, *pgt*P, *hly*F, the *pix* gene cluster and *fec*R (Fig. 3).

### Analysis of the capsular polysaccharide synthesis gene cluster

**Fig. 4:**
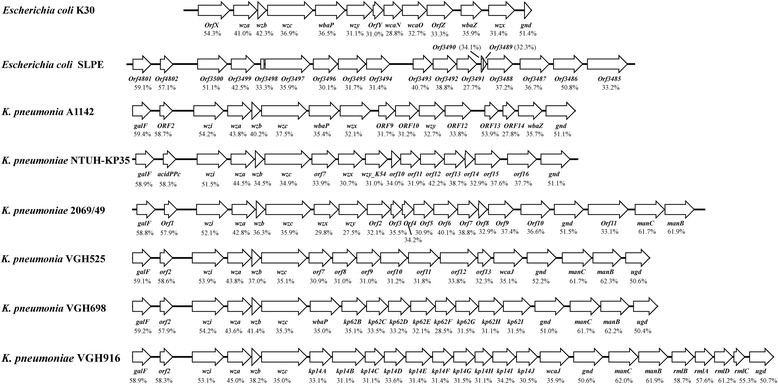
The genetic context for seven different types of capsular polysaccharide synthesis gene clusters. Shaded areas show conservative genes. Arrows represent the coding sequences and indicate the direction of transcription. The percentage under arrow represents the G/C content of the gene

We found a capsular polysaccharide synthesis gene cluster (CPS) in the SLPE genomes using blastp. This CPS was closely related to the *Klebsiella pneumonia* CPS. The *Klebsiella pneumonia* CPS is closely related to *Klebsiella pneumonia* pathogenesis. The similarity and GC content of the 5 CPSs from *Klebsiella pneumoniae* genomes and 1 CPS from an *E. coli* strain were analysed. The results are shown in Fig. 4.

## Discussion

In this study, we isolated ExPEC isolates (ALPE) from pig farms and demonstrated that the SLPE isolates are pneumonia pathogens. Then, we performed genome sequencing and comparative genomic analysis with the SLPE isolates. Our analysis identified differences between SLPE and other ExPEC strains including various differences in the toxin and capsular polysaccharide synthesis gene clusters.

The phylogenomic comparison indicated that the SLPE strain belongs to the B1 monophyletic phylogroup, with its closest relative being APEC O78. APEC O78, an O78 strain, is an avian pathogenic *E. coli* isolated from the lungs of turkey that caused extensive animal and financial losses globally [6]. Although the SLPE and APEC O78 hosts are different, the strains have similar evolutionary relationships and both contain 4033 homologous genes. Now the question remains whether they have a common ancestor that is worthy of our attention.

ExPEC strains contain certain specific virulence traits that enable them to invade and colonize extraintestinal sites and cause a wide range of infections [7]. In this study, the distribution diagram of the pan-genome virulence genes showed that there were significant differences between the SLPE and APEC078, although they are most closely related to one another. We also found that SLPE has more ExPEC-specific virulence factors, including several virulence factors that are ExPEC strain-specific, such as fimbrial adhesins (*pap*G II), *ire*A, *pgt*P, *hly*F, the *pix* gene cluster and *fec*R. Of these genes, only *pap*G II has been proven to have contact with urethral and avian pathogenicity [8]. These genes’ functions in pig ExPECs have yet to be explored.

In China, Liu et al., has reported the full genome characterization of ExPEC PCN033 strain isolated from pigs. The ExPEC virulence markers are different between PCN033 and SLPE, and the comparative genomic analysis results are different. SLPE was assigned to group B1 and clustered with APEC 078, while PCN033 was assigned to group D1 [9]. Furthermore, we found a capsular polysaccharide synthesis gene cluster (CPS) in the SLPE strain genomes, this CPS was closely related to the Klebsiella pneumonia CPS. The gene homology was approximately 96% (*wzi*, *wza* and *wzc*) and the homology of genes responsible for the serotype (*wzx* and *wzy*) was approximately 99%. Additionally, the GC content of the CPS was 51.7%, which is significantly lower than the full genome. These results suggest that the SLPE CPS may have been transferred from *Klebsiella pneumoniae*. Based on further analysis, the *Klebsiella pneumonia* CPS is the best characterized virulence factor in pneumonia and urinary tract infections [10, 11]. Functional analysis of the SLPE CPS will be forthcoming.

## Conclusions

The SLPE isolated from swine lungs in China, was assigned to group B1 and clustered with APEC 078. The comparative genomic analysis revealed several genes for their further study. We found a CPS in the SLPE strain genomes, and this CPS is closely related to the CPS from *Klebsiella pneumoniae*. In conclusion, the mechanisms of ExPEC virulence still remain largely unknown. Studies on ExPEC pathogenesis will be enhanced by public access to high-quality genomic sequences.

## Methods

### Gross post-mortem examination

The swine industry was affected by pneumonia in the Changchun district of China in 2014. The pigs presented with pneumonia for 4–6 days prior to death. The autopsy performed after death included gross examination and observations of the lesions according to standard operating procedures. Lung tissue samples were quickly removed and fxed in formalin 10%. The diagonal section of samples was obtained and sectioned at 4 μm thickness, then the samples were stained using hematoxylin and eosin(H&E) stains, assessed using a light microscopy.

### Bacterial isolation, culture conditions and serogroup

Bacteria were isolated from the surface of heat sterilized tissues, including lung, lymph, brain and heart blood samples.. All isolates were cultured on Mueller Hintom (MH), MacConkey, chocolate and 5% sheep’s blood agar plates. The isolates were identified by 16S rRNA gene sequence analysis and all isolates were identified in PCR for virulence marker genes of ExPEC (papA/papC, sfa/foc, afa/dra, kpsMTII and iutA) and ExPECs were defined as the isolates containing two or more virulence markers. Then, the serogroup was determined using antisera from the China Institute of Veterinary Drug Control (Beijing, China).

### Experimental challenge studies

To test the pathogenicity of the porcine ExPEC strains, Experimental challenge studies was performed. The protocol was approved by the Committee on the Ethics of Animal Experiments of the Jilin Agricultural University. A total of 20 BALB/c mice weighing 20(±2) g were randomly divided into 4 groups, every group contained 5 mice (purchased from the Animal Centre of Jilin University, Changchun, China) according to the institutional guidelines for the use of experimental animals (Laboratory animal─requirements of environment and housing facilities number GB 14925–2010). Approximately 5 × 10^7^ CFU, 5 × 10^6^ CFU and 5 × 10^5^ CFU SLPE suspensions were prepared, and the bacterium were centrifuged and cleaned by physiological saline. Then, the strains were injected intraperitoneally into BALB/c mic, physiological saline was used as a negative control. Mice for health status were observed twice daily during 3 days. Deaths were recorded, and histological examination of dead mice lung was performed.

### Antimicrobial resistance

Antimicrobial susceptibility testing was performed using the microplate dilution method [12]. The antimicrobial agents included cefotaxime, florfenicol, doxycycline, ciprofloxacin, tilmicosin, enrofloxacin, sulfamethoxazole and amikacin. The reference strain *E. coli* ATCC 25922 served as a standard for quality control. Each experiment was repeated four times.

### DNA extraction

Total DNA was extracted using the Rapid Bacterial Genomic DNA Isolation Kit (Sangon Biotech, Shanghai, China) and stored at −80 °C until use. The DNA preparation was used for genome sequencing.

### Genome sequencing and genome assembly

A whole genome shotgun library was produced with 1 μg of genomic DNA according to the instructions of the TruSeq™ DNA Sample Prep Kit-Set A (Illumina, USA) and the TruSeq PE Cluster Kit (Illumina, USA). The libraries were sequenced on an Illumina MiSeq platform at the Laboratory of Chinese National Human Genome Center (Shanghai, China). Afterward, the sequences were assembled using the SOAPdenovo software package [13, 14]. A total of 9,569,782 paired-end reads were assembled into 336 contigs.

### Gene prediction and functional annotation

Coding sequences were predicted by combining the results obtained with the glimmer 3.02, Genemark and Z-Curve programs [15, 16]. Translational products of the coding sequences were annotated with the GenBank database. Putative functions of translation products were confirmed using the KEGG and Clusters of Orthologous Groups (COGs) databases [17, 18]. Putative genes of interest were identified with NCBI ORF finder and iterative BLASTn and BLASTp searches.

### Phylogenomic comparison of SLPE with other *E. coli* pathotypes

Forty-three *E. coli* strain genomes were downloaded from the NCBI GenBank, fourteen strains was ExPEC, belong to different groups. The common genes were considered by the BLAST-like alignment tool (e-value = 1-e^−10^) and mcl [19]. All of the common genes were aligned by MUSCLE and concatenated together [20]. Then, clustalW and MEGA were used to reconstruct the phylogenomic tree.﻿ Genomes of Escherichia coli used for phylogenetic construction and core genome content used to construct phylogenetic tree were listed in Additional file 1: Table S1 and Additional file 2: Table S2.﻿

### The distribution diagram of pan-genome virulence genes

To examine whether SLPE strains harboured unique ExPEC-specific virulence factors, the pan-genome analysis of 51 virulence factors were performed, which are all positive in SLPE isolate [21]. ExPEC-specific virulence factors were classified into the following six categories: (1) adhesins, (2) invasins, (3) toxins, (4) iron acquisition/transport systems, (5) polysialic acid synthesis and (6) other virulence genes. All of the virulence factors were conducted with blastp and the virulence genes were clustered with PGAP. Then, a distribution diagram was created with the R program (http://www.r-project.org/). Six groups of genes that were used for heat map construction were listed in Additional file 1: Table S1 and Additional file 3: Table S3.

### Analysis of capsular polysaccharide synthesis gene cluster

We found a CPS in the genome of the SLPE strain using blastp. The SLPE CPS was a close relative to the CPS from *Klebsiella pneumoniae* in the NCBI GenBank. The *Klebsiella pneumoniae* CPS is considered to be closely related to pathogenesis. Therefore, analysis of the CPS gene cluster between SLPE and other *Klebsiella pneumoniae* was important. Six CPSs from *Klebsiella pneumoniae* genomes and 1 CPS from an *E. coli* strain were downloaded from the NCBI GenBank. The GC content was calculated by GC content.pl and analysed with clc sequence viewer. Then, the similarity was analysed by mauve. CPS gene clusters were listed in Additional file 4: Table S4.

## Additional files

Additional file 1: Table S1. Genomes of *Escherichia coli* used for phylogenetic construction. (DOC 135 kb)

Additional file 2: Table S2. Core genome content used to construct phylogenetic tree. (XLSX 18 kb)

Additional file 3: Table S3. Six groups of genes that were used for heat map construction. (DOCX 16 kb)

Additional file 4: Table S4. Comparison of CPS gene cluster of SLPE with closely related CPS gene clusters from other genomes. (DOCX 14 kb)

## Abbreviations

APEC: Avian pathogenic *Escherichia coli*
CPS: Capsular polysaccharide synthesis
*E. coli*: *Escherichia coli*
ExPEC: extraintestinal pathogenic *Escherichia coli*
