# Supplementary material for: Whole genome sequencing of an ExPEC that caused fatal pneumonia at a pig farm in Changchun, China
Source: BMC Vet Res. 2017 Jun 9;13:169. doi: 10.1186/s12917-017-1093-5 (PMC5466758; doi:10.1186/s12917-017-1093-5)
Supplement: Supplementary file 1 — Genomes of Escherichia coli used for phylogenetic construction. (DOC 135 kb) [file 12917_2017_1093_MOESM1_ESM.doc]

Table S1. Genomes of Escherichia coli used for phylogenetic construction

| **No.** | **Strains（Escherichia coli）** | **Origin** | **GenBank Accession No.** |
| --- | --- | --- | --- |
| 1 | ATCC 8739 | non-pathogenic | NC_010468 |
| 2 | MG1655 | non-pathogenic | NC_000913 |
| 3 | UMNK88 | ETEC | NC_017641 |
| 4 | 2.3916 | STEC | NZ_[AFAB00000000](http://www.ncbi.nlm.nih.gov/Traces/wgs/?val=AFAB02#contigs) |
| 5 | PCN033 | ExPEC/**NMEC** | NZ_[AFAT00000000](http://www.ncbi.nlm.nih.gov/Traces/wgs/?val=AFAT01#contigs) |
| 6 | APEC O78 | ExPEC/**APEC** | NC_020163 |
| 7 | APEC O1 | ExPEC/**APEC** | NC_008563 |
| 8 | IAI39 | ExPEC/**UPEC** | NC_011750 |
| 9 | S88 | ExPEC/**NMEC** | NC_011742 |
| 10 | O157：H7 sakai | EHEC | NC_002695 |
| 11 | E2348/69 | EPEC | NC_011601 |
| 12 | 55989 | EAEC | NC_01I748 |
| 13 | E24377A | ETEC | NC_009801 |
| 14 | IAI1 | non-pathogenic | NC_011741 |
| 15 | KO11FL | The engineering bacteria | CP002970 |
| 16 | O103:H2 str. 12009 | EHEC | NC_013353 |
| 17 | O104:H4 | EAEC | NC_018658 |
| 18 | 11128 | EHEC | NC_013364 |
| 19 | 11368 | EHEC | NC_013361 |
| 20 | BL21 | The engineering bacteria | NC_012947 |
| 21 | BW2952 | laboratoiy-adapted | NC_012759 |
| 22 | CB9615 | EPEC | NC_013941 |
| 23 | CE10 | ExPEC/**NMEC** | NC_017646 |
| 24 | CFT073 | ExPEC/**UPEC** | NC_004431 |
| 25 | DHl | - | NC_017625 |
| 26 | E234869 | EPEC | NC_011601 |
| 27 | NRG857C | AIEC | NC_017634 |
| 28 | ABU83972 | ExPEC/**UPEC** | NC_017631 |
| 29 | 536 | ExPEC/**UPEC** | NC_008253 |
| 30 | IHE3034 | ExPEC/**NMEC** | NC_017628 |
| 31 | EDL933 | EHEC | NC_002655 |
| 32 | HS | non-pathogenic | NC_009800 |
| 33 | Xuzhou21 | EHEC | NC_017906 |
| 34 | RM12579 | EPEC | NC_017656 |
| 35 | TW14359 | EHEC | NC_013008 |
| 36 | LF82 | AIEC | NC_011993 |
| 37 | REL606 | The engineering bacteria | NC_012967 |
| 38 | SMS-3-5 | non-pathogenic | NC_010498 |
| 39 | UMN026 | ExPEC/**UPEC** | NC_011751 |
| 40 | UTI189 | ExPEC/**UPEC** | NC_007946 |
| 41 | SE11 | - | NC_011415 |
| 42 | 042 | EAEC | NC_017626 |
| 43 | HI0407 | ETEC | NC_017633 |

Strains with underlines are previously reported ExPECs
