# Supplementary material for: Whole genome sequencing of an ExPEC that caused fatal pneumonia at a pig farm in Changchun, China
Source: BMC Vet Res. 2017 Jun 9;13:169. doi: 10.1186/s12917-017-1093-5 (PMC5466758; doi:10.1186/s12917-017-1093-5)
Supplement: Supplementary file 3 — Six groups of genes that were used for heat map construction. (DOCX 16 kb) [file 12917_2017_1093_MOESM3_ESM.docx]

Table S3. six kinds of genes for drawing hot map

| fimbrial/adhesin | |  |
| --- | --- | --- |
| ORF | name | function |
| ORF_2999 | papG | p fimbrialadhesin |
| ORF_3276 | fimH | type 1 fimbrial adhesin |
| ORF_4158 | fimH | D-mannose specific adhesin |
| ORF_1824 | upaG/ehaG | autotransporter proteins |
| ORF_3048 | eaeH | Putative adhesin |
| ORF_0889 | ydeQ | putative fimbrial-like exported adhesin protein |
| ORF_1057 | yadN | fimbrial-like adhesin protein |
| ORF_1065 | yadC | putative fimbrial-like adhesin protein |
| ORF_2058 | yfaL | adhesin |
| ORF_2378 | ybgD | putative fimbrial-like adhesin protein |
| ORF_4681 | fimF | fimbrial-like adhesin protein |
| ORF_0894 | focA | S fimbrial adhesin major subnit sfaA |
| ORF_4567 | papC | Outer membrane usher protein PapC |
| ORF_2914 | papC | outer membrane usher protein PapC precursor |
| ORF_2913 | papH | minor pilin protein PapH |
| ORF_2912 | papA | PapA protein |
| ORF_3041 | ecpD | CFA/I fimbrial minor adhesin |
| ORF_1824 | YadA | hemagglutinin family protein |
| ORF_4764 | pgaB | biofilm adhesin polysaccharide PGA export lipoprotein with a polysaccharide deacetylase activity needed for export |
| Iron acquisition/exporting protein | | |
| ORF | name | function |
| ORF_4352 | ireA | Putative iron-regulated outer membrane virulence protein |
| ORF_4164 | iutA | TonB-dependent siderophore receptor |
| ORF_4168 | iucA | Aerobactin siderophore biosynthesis protein |
| ORF_3872 | iroN | iron-related receptor of siderophore salmocheline |
| ORF_3869 | iroC | salmochelin export ABC transporter ATP-binding protein |
| ORF_2098 | fepC | ATP-binding component of ferric enterobactin transport |
| ORF_2092 | fepA | outer membrane receptor for ferric enterobactin |
| ORF_2868 | fyuA | pesticin/yersiniabactin TonB-dependent receptor |
| ORF_2863 | irp2 | yersiniabactin biosynthetic protein |
| ORF_2864 | irp1 | yersiniabactin biosynthetic protein |
| ORF_4171 | sitD | Iron transport protein |
| ORF_4172 | sitC | chelated iron transport system membrane protein |
| ORF_4173 | sitB | iron transport protein, ATP-binding component |
| ORF_4174 | sitA | iron transport protein, periplasmic-binding protein |
| ORF_0187 | fecR | iron dicitrate transport regulator |
| toxin/Invasin | |  |
| ORF_4775 | hlyF | hemolysin F |
| ORF_2998 | tia | Tia invasion determinant |
| ORF_2079 | ibeB | copper/silver efflux system outer membrane protein CusC |
| ORF_2011 | aslA | Arylsulfatase (EC 3.1.6.1) |
| ORF_3896 | ClpV1 | type VI secretion ATPase |
| ORF_3890 | hcp | Hcp-like protein |
| ORF_0714 | vgrG | VgrG protein |
| ORF_0814 | cirA | colicin I receptor |
| Capsule synthetic gene cluster | | |
| ORF_3495 | wzy-k16 | serotype K16 polymerase |
| ORF_3493 | orf11 | putative glycosyltransferase |
| ORF_3494 | orf10 | group 1 glycosyl transferase |
| ORF_3492 | orf12 | GDP-mannose 4,6-dehydratase |
| Protectins | |  |
| ORF_0144 | ompW | Cell envelope biogenesis, outer membrane |
| ORF_4526 | traT | conjugal transfer surface exclusion protein TraT |
| ORF_1602 | cvpA | colicin V production protein |
| others |  |  |
| ORF_0983 | MalX | PTS system, maltose and glucose-specific IIBC component |
| ORF_3103 | ybjX | esterase |
| ORF_1560 | dsdA | d-Serine deaminase |
| ORF_2907 | pgtP | transporter protein |
