# Supplementary material for: Whole genome sequencing of an ExPEC that caused fatal pneumonia at a pig farm in Changchun, China
Source: BMC Vet Res. 2017 Jun 9;13:169. doi: 10.1186/s12917-017-1093-5 (PMC5466758; doi:10.1186/s12917-017-1093-5)
Supplement: Supplementary file 4 — Comparison of CPS gene cluster of SLPE with closely related CPS gene clusters from other genomes. (DOCX 14 kb) [file 12917_2017_1093_MOESM4_ESM.docx]

Table S4. The gene cluster for cps compare and analysis

| Strains | serotype | Cps cluster Accession No. | ORF No. |
| --- | --- | --- | --- |
| *Escherichia coli* K30 | K30 | AF104912 | 14 |
| Klebsiella pneumoniae 2069/49 | K16 | AB742228 | 21 |
| Klebsiella pneumoniaeVGH916 | K14 | AB371294 | 25 |
| Klebsiella pneumonia VGH525 | K2 | AB371296 | 18 |
| Klebsiella pneumoniaVGH698 | K62 | AB371295 | 19 |
| Klebsiella pneumoniaAB334776 | K57 | AB334776 | 16 |
| Klebsiella pneumoniaeNTUH-KP35 | K54 | AB289650 | 17 |
